# Teaching biotechnology through practical cases

**DOI:** 10.1186/1753-6561-8-S4-P229

**Published:** 2014-10-01

**Authors:** Bastos Teodiano

**Affiliations:** 1 Universidade Federal do Espírito Santo (UFES), Vítoria, ES, Brazil

## Background

A good way to teach biotechnology is showing practical cases after explaining the theory.

## Methods

Three cases of study and application of biomedical signals are analyzed:

a) Development of a multisensorial prosthesis of upper limb commanded by myoelectric signals (sEMG)

b) Development of a robotic wheelchair commanded by eye blinks (myoelectric signals - sEMG) and by brain signals (EEG)

c) Use of myoelectric signals (sEMG) from disabled children to interact with mobile robots in order to carry out actions in the environment

In the first one, students are motivated to understand how a myoelectric signal is produced and then this knowledge is applied in upper limb prosthesis.

In the second case, brain signals are also explained. Then ERD/ERS (Event Related Desynchronization /Event Related Synchronization) in alpha rhythm and Steady State Visual Evoked Potential (SSVEP) are used to command a robotic wheelchair. Myoelectric signals are also used to command the wheelchair; in this case, these signals are captured from the user face due to eye blinks.

Finally, for the third case, myoelectric signals are captured from disabled children to allow them to interact with a mobile robot, in order to the mobile robot carrying out movements of grasping objects using its tweezers, paint, etc.

## Results

Several videos of experiments about the three cases are shown to students and discussions about the strategy of using the methodology of "hands on" when teaching biotechnology are exposed, based on these practical cases.

